# Involvement of mast cell chymase in burn wound healing in hamsters

**DOI:** 10.3892/etm.2012.836

**Published:** 2012-11-27

**Authors:** XIANGLIN DONG, ZHONGLI GENG, YANG ZHAO, JUNJIE CHEN, YING CEN

**Affiliations:** 1 Department of Burns and Plastic Surgery, The First Affiliated Hospital of Xinjiang Medical University, Urumqi, Xinjiang Uigur Autonomous Region 830054;; 2 Department of Breast, Head and Neck Surgery, Affiliated Tumour Hospital, Xinjiang Medical University, Urumqi, Xinjiang Uigur Autonomous Region 830011;; 3 Department of Burns and Plastic Surgery, West China Hospital, Sichuan University, Chengdu, Sichuan 610041, P.R. China

**Keywords:** chymase, mast cell, Ang II, TGF-β1, IL-1β

## Abstract

Mast cells play a significant role in the late stage of wound healing following burn injuries. In the present study, the possible role of mast cell chymase in burn wound healing was examined using a mast cell membrane stabilizer, ketotifen, in hamsters. A total of 28 hamsters were randomly divided into two groups (n=14), termed as the control and ketotifen groups. A deep partial-thickness burn injury was made on the back skin of the hamsters. The control group was orally administered physiological saline (1 ml) and the ketotifen group was orally administered ketotifen (4 mg/kg) once daily, two days prior to and two days subsequent to the burn. The results showed that concentrations of angiotensin II (Ang II), TGF-β1, collagens I and III and interleukin (IL)-1β were significantly decreased in the ketotifen group compared with those in the control group. However, there was no significant difference in fibroblast apoptosis between the two groups. The release of mast cell chymase was inhibited by the mast cell membrane stabilizer ketotifen. Taken together, these results suggest that mast cell chymase may participate in the process of burn wound healing. Chymase may therefore be a promising therapeutic target for the treatment of burn wounds.

## Introduction

Burn wounds usually result in tissue ischemia and inflammation, leading to increased numbers of mast cells (1,2). Mast cells play a significant role not only in the acute inflammatory phase but also in the late stage of wound healing following burn injury (3). Chymase is an enzyme that is mainly located in mast cell granules. The enzyme has previously been demonstrated to be activated in tissue fibrosis, including pulmonary fibrosis (4,5) and cardiac fibrosis (6). Significantly, the involvement of chymase in tissue matrix remodeling has been suggested by its ability to activate procollagenase (7) and degrade the extracellular matrix (ECM) (8). One of the major functions of chymase is to convert angiotensin I (Ang I) to angiotensin II (Ang II). Moreover, chymase contributes to the release of TGF-β1 from its precursor, human fibroblast latent TGF-β1-binding protein (9). Certain studies also showed that chymase is able to convert inactive interleukin-1β (IL-1β), a proinflammatory cytokine, to its active form (10,11).

Ang II is the major effector peptide in the renin-angiotensin system (RAS). Besides being a physiological mediator restoring circulatory integrity (12,13), Ang II has been recognized as a growth factor that regulates cell growth, angiogenesis, inflammation, tissue repair and remodeling (2). Ang II in the human heart is generated via two pathways, the angiotensin converting enzyme (ACE) pathway and the chymase pathway. The chymase pathway accounts for ∼80% of Ang II formation in the heart (14). Similar pathways also exist in hamsters (15,16).

A previous study showed that mast cell chymase played a key role in the normal wound healing process by measuring the size of the burn wounds, the density of the capillaries, collagen accumulation, mast cell number and chymase activity in the mouse dorsal skin prior to and 1, 3, 7 and 14 days subsequent to burning (17). However, the role of ACE-independent production of Ang II by the chymase enzyme in burn injuries remains unclear.

In the present study, in order to investigate the role of mast cell chymase in burn wounds, the mast cell membrane stabilizer ketotifen was orally administered to hamsters with partial-thickness burn injuries. The levels of Ang II in the mast cells from the burn tissues were analyzed. Meanwhile, TGF-β1 and IL-1β levels were also examined. Moreover, as collagen is the main component of the ECM, the expression of this protein was also investigated to assess the healing of burned tissues.

## Materials and methods

### Animal experiment

In total, 28 eight-week-old hamsters were purchased from the Urumuqi Center for Disease Control and Prevention in the Xinjiang Uyghur Autonomous Region of China. Animals were housed in individual stainless steel cages in a temperature-controlled environment (25–30°C) with 12 h light-dark cycles. Food pellets and water were available *ad libitum*. Animals were acclimatized for a minimum of 2 weeks prior to thermal injury. All animal care and experimental protocols were approved by the Animal Ethics Committee of the First Affiliated Hospital of Xinjiang Medical University and were in accordance with institution guidelines.

### Burn wounds in hamsters

The 28 eight-week-old hamsters were randomly divided into two groups. Half of the hamsters (n=14) formed the control group and the other half (n=14) formed the ketotifen group. The animals were weighed and then anesthetized with ketamine (0.7 g/kg) i.p. Diazepam and atropine were added to maintain adequate anesthesia. Once anesthetized, the dorsal torso of each animal was shaved and a commercial depilating agent was applied to fully remove the hair in an ∼3 cm diameter area. The control and ketotifen groups were orally administered physiological saline (1 ml) and ketotifen (4 mg/kg), respectively, once daily for two days prior to burning and for two days subsequent to burning.

A scald template was fashioned from the caudal end of a plastic 50 ml syringe without the plunger. The caudal end was placed on the dorsal torso skin of hamster with a gentle pressure that just kept the water in the syringe. Then 20 ml of 75°C water was put into the 50 ml syringe without the plunger to create a scald wound of ∼3 cm in diameter with a contact time of 12 sec. The burn area covered ∼5% of the total body surface. A deep partial-thickness burn injury was made on the back skin in this pattern with high reproducibility. Every hamster from the two groups survived the process. The hamsters were sacrificed and the back skin was harvested on day 3 subsequent to burning.

### Measurement of Ang II

The quantitative measurement of Ang II in burn tissues was measured by a radio-immunity kit (Beijing North Institute of Biological Technology, Beijing, China). A total of 0.1 g burn tissues was minced and homogenized subsequent to being washed in cold saline. The burn tissue was transferred into a tube containing 1 ml NaCl (0.9%) and centrifuged at 12,000 × g for 15 min. The supernatant was used to measure Ang II levels via a radioimmunoassay (RIA). The RIA for Ang II was performed using 125I-angiotensin II and rabbit anti-ANG II antibody (Beijing North Institute of Biological Technology) in accordance with the instructions of the radio-immunity kit. The ratio of B/Bo (B, experimental condition; Bo, control condition) was corrected for non-specific binding, presented as a percentage of maximal binding and read against a standard curve (log-logit transformation).

### Flow cytometry

A section of the burn tissue was cut into 1x1-mm samples subsequent to being rinsed and was then homogenized with cold Hanks’ balanced salt solution. The mixed tissue fluid was placed on a filter of 300 mesh and then the filtered liquid was centrifuged at 2,000 × g for 5 min. Cells were collected and suspended in Hanks’ balanced salt solution, counted with a hemocytometer and adjusted to a concentration of 10^6^ cells/ml. A quantitative assessment of apoptosis was performed using the Annexin V-FITC apoptosis detection kit as described by the manufacturer’s instructions (Kaiji Bio Co., Nanjing, China; Cat. #, KGA108). Briefly, cells were treated with 5 *μ*l Annexin V-FITC and 5 *μ*l propidium iodide (PI) and placed in the dark at room temperature for 15 min prior to being run through the flow cytometer. Data were acquired on a Beckman Coulter XL (Beckman Coulter, Fullerton, CA, USA).

### Western blotting analysis

Total protein from the burn tissues of the hamsters was extracted with protein lysate buffer containing PMSF (1 mmol/l) following homogenization. Tissue lysates were centrifuged at 12,000 × g for 5 min at 4°C. Protein concentrations in the supernatant from each group were determined by using a BCA protein quantitative analysis kit (BMD Biomed Tech, Beijing, China). An equal amount (4 *μ*l) of protein from each supernatant was subjected to 8–10% gradient sodium dodecylsulfate-polyacrylamide gel electrophoresis. Following electrophoresis, proteins were transferred onto a polyvinylidene fluoride (PVDF) membrane (Invitrogen, Carlsbad, CA, USA). The PVDF membrane was then incubated for 1 h at 4°C with the primary antibody following block solution treatment. The primary antibodies used in this study were mouse anti-TGF-β1, mouse anti-collagens I and III, mouse anti-IL-1β and mouse anti-β-actin (1:100; Santa Cruz Biotechnology, Santa Cruz, CA, USA). Following incubation with the primary antibody, the membranes were probed with the appropriate alkaline phosphatase-conjugated secondary antibody (anti-mouse or anti-rabbit, 1:20,000; Invitrogen) and then incubated with a solution of BCIP/NBT substrate for alkaline phosphatase until the appearance of the purple band. An efficient transfer was confirmed by staining the membrane with Ponceau S. The relative intensity of the immunoreactive bands was quantified using a computer-assisted densitometry program (BioRad Tech, Hercules, CA, USA).

### Statistical analysis

The results are shown as mean ± standard deviation (mean ± SD). An analysis of variance and Dunnett’s t-test were performed to evaluate the differences between groups, using SPSS 10.0 software (Madison, WI, USA). Statistical differences were considered significant if P<0.05.

## Results

### Ang II levels were significantly decreased in the ketotifen group

**Figure 1.**
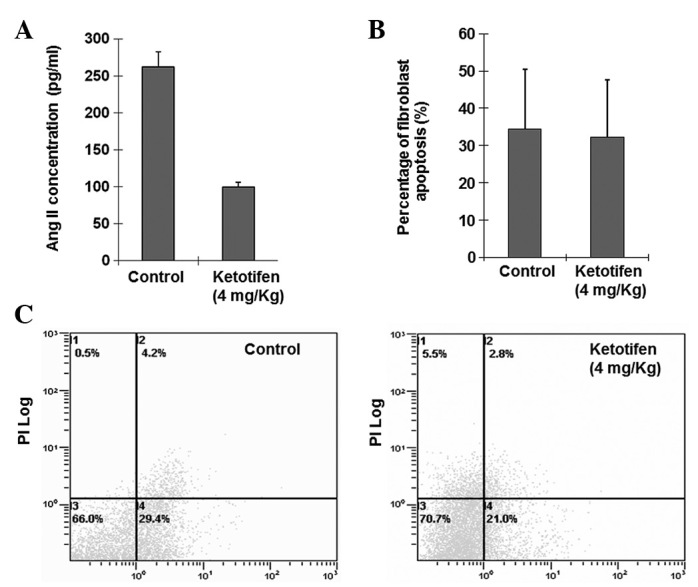
Change of angiotensin II (Ang II) concentration in burn tissues and fibroblast apoptosis. (A) The ketotifen group was orally administered ketotifen (4 mg/kg) once daily, two days prior to and two days subsequent to the burn. Control hamsters were treated with saline (1 ml). Ang II concentrations in the two groups were measured as described in Materials and methods. Ang II concentration (mean ± SD) in the ketotifen group was significantly lower than that in the control group (n=14). (B) Fibroblast apoptosis rates of the burn tissues. There was no significant difference between the ketotifen and control groups (P>0.05). (C) Representative examples of fibroblast apoptosis of the burn tissue cells using the Annexin V-FITC/PI-stained fluorescence-activated cell sorter (FACS) analysis.

There was a significant difference in Ang II levels in burn tissues between the two groups (P<0.05). Fig. 1A shows that Ang II levels in burn tissues were significantly decreased in the ketotifen group (100.1142±6.0702 pg/ml) when compared with the control group (261.8450±20.8356 pg/ml). Therefore, the results suggest that chymase converted Ang I to Ang II in burn wound tissues.

### Fibroblast apoptosis rates were similar between the ketotifen group and the control group

Alterations of the plasma membrane, with translocation of phosphatidylserine from the inner side of the plasma membrane to the external surface, are the hallmark of apoptosis. The Annexin V-FITC/PI-stained fluorescence-activated cell sorter (FACS) analysis of fibroblast apoptosis in the burn tissues indicated that the percentage of apoptotic cells (34.4±16.05 versus 32.32±0.1534%) were similar (P>0.05; Fig. 1B and C) in the two groups.

### Ketotifen significantly suppresses collagen accumulation in the burn wound

**Figure 2.**
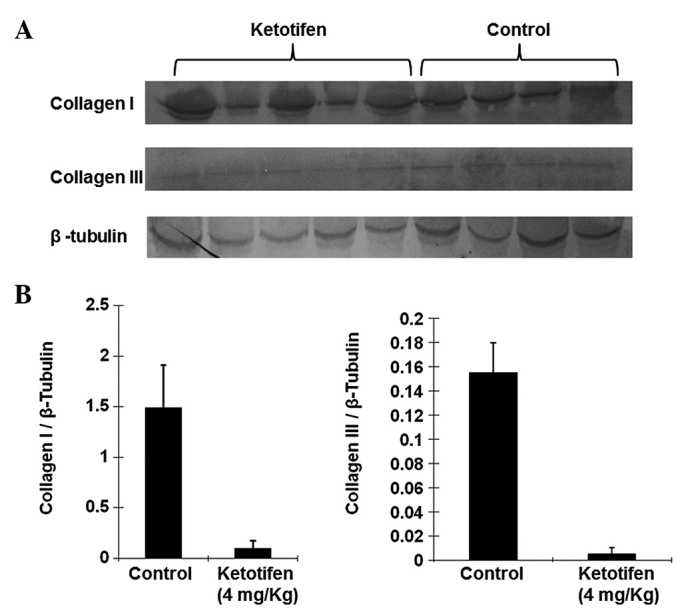
Expression levels of collagen I and III in burn tissues. (A) Western blot analysis for the expression of collagen I and III proteins in burn tissues. Western blot analysis showed a clear signal at ∼90 kDa (collagen I) and 190 kDa (collagen III) for the two groups. The control group showed a stronger expression of collagen I and III proteins than the ketotifen group. A strong ∼43 kDa signal for β-tubulin was observed for all tissues tested. (B) According to the western blot analysis, the ketotifen group had significantly lower expression levels of collagens I and III than that of the control group. The expression levels were determined as a ratio of collagen I or III to β-tubulin to correct the variation in the protein quantity.

Collagen is the main component of the ECM. Levels of collagen I and III in the burn tissues of the two groups were markedly different. Fig. 2 shows that the expression levels of collagen I and III relative to those of b-tubulin in burn tissues from the ketotifen group (collagen I, 0.1013±0.0755; collagen III, 0.0054±0.0051) were significantly lower than those of the control group (collagen I, 1.4903±0.4230; collagen III, 0.1548±0.0248; P<0.01). This result suggested that ketotifen was able to significantly suppress collagen accumulation in the burn wound.

### Ketotifen inhibits the chymase-induced generation of mature TGF-β1 in burn wounds

**Figure 3.**
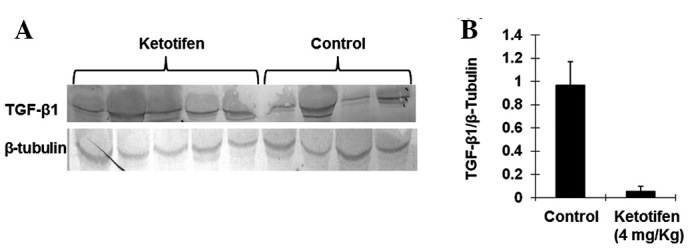
Expression of TGF-β1 in burn tissues. (A) Western blot analysis for the expression of TGF-β1 protein in burn tissues. A clear signal was observed at ∼25 kDa (TGF-β1) for the two groups. (B) Compared with the control group, the expression of TGF-β1 in the ketotifen group was significantly decreased. The expression levels were determined as a ratio of TGF-β1 to the reference protein β-tubulin to correct for the variation in protein quantity.

TGF-β1 is thought to be one of the major cytokines involved in organ fibrosis. The level of TGF-β1 was therefore also investigated by western blotting. As shown in Fig. 3, there was a significant difference in the TGF-β1 expression levels between the ketotifen group (0.0518±0.0449) and the control group (0.9645±0.2046). The expression of TGF-β1 was significantly decreased in the ketotifen group (P<0.01). This result suggested that ketotifen inhibited the chymase-induced generation of mature TGF-β1 in burn wounds. Thus, chymase may contribute to TGF-β1 activation.

### Ketotifen treatment markedly decreased the expression of IL-1β

**Figure 4.**
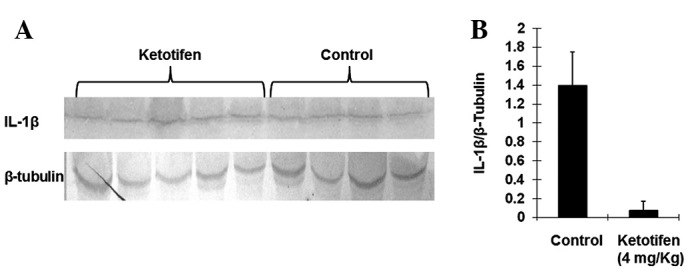
Expression of interleukin (IL)-1β in burn tissues. (A) Western blot analysis for the expression of IL-1β protein in burn tissues. A clear signal was observed at ∼31 kDa (IL-1β) for the two groups. (B) Compared with the control group, the expression of IL-1β in the ketotifen group was decreased significantly. The expression levels were determined as a ratio of IL-1β to the reference protein β-tubulin to correct for the variation in the protein quantity.

IL-1β is a significant proinflammatory factor. Mast cell chymase is able to induce specific conversion of the IL-1β precursor to an active IL-1 species in humans (18). As shown in Fig. 4, ketotifen treatment markedly decreased the expression of IL-1β (0.0740±0.0945) as compared with the control group (1.3913±0.3853), which indicated that mast cell chymase may be involved in the activation of IL-1β in burn tissues (P<0.05).

## Discussion

It has previously been shown that mast cell chymase is the Ang II forming enzyme in the major non-ACE pathway in the heart (19). Such pathways were later identified not only in the human heart, but also in the thoracic artery, saphenous vein (20), radial artery (21), gastroepiploic artery (22), bleomycin-induced pulmonary fibrosis (4,5,23) and cardiac fibrosis (6).

In the present study, we used ketotifen, a mast cell membrane stabilizer, to investigate the production of Ang II in burn tissues in hamsters. The results showed that the production of Ang II in the ketotifen group was significantly decreased. This suggests that mast cell chymase has the same effects on the conversion of Ang I to Ang II in burn tissues as it does in other tissues or organs, including the heart (6).

Ang II is the major effector peptide in the RAS. Besides being a physiological mediator restoring circulatory integrity (12,13), Ang II is now recognized as a growth factor that regulates cell growth, angiogenesis, inflammation, tissue repair and remodeling (2). Ang II contributes greatly to tissue fibrosis, including hepatic (24), renal (25) and cardiac fibrosis (26). Ang II combines with Ang II type-1 receptor to increase the expression of TGF-β1 (27,28). In addition, chymase also contributes to the release of TGF-β1 from its precursor (9). TGF-β1 has been identified as the most significant profibrotic cytokine (29), it induces an increase in collagen production and secretion and enhances the abundance of mRNA levels for collagen types I and III (30). Ang II also activates collagen I gene expression, but would require activation of the MAPK/ERK and TGF-β signaling pathways (31).

The present study showed that ketotifen treatment significantly reduced the production of TGF-β1 and collagens I and III. These results indicate that greatly decreased Ang II levels cannot induce excessive expression of TGF-β or collagen I and III genes due to the deficiency in activated mast cell chymase.

Ang II is able to stimulate not only cardiac fibroblast proliferation (32) but also skin fibroblast proliferation (33). Certain studies have indicated that mast cell chymase is able to induce smooth muscle cell and endothelial cell apoptosis (34–36). However, no studies have reported whether or not Ang II and chymase are able to induce fibroblast apoptosis. The present study showed that there was no significant difference in fibro-blast apoptosis between the ketotifen group and the control group. This result indicates that mast cell chymase may have no effect on fibroblast apoptosis.

According to previous studies Ang II had no effect on the the activation of IL-1β. However, human mast cell chymase is able to induce the accumulation of neutrophils, eosinophils and other inflammatory cells *in vivo*(37,38), as well as the rapid and specific conversion of precursor IL-1β to an active IL-1 species (39). In the present study, in comparison to the control group, IL-1β was greatly reduced in the ketotifen group, suggesting that chymase may be involved in the activation of IL-1β in burn tissues.

Wound healing subsequent to burn injuries is an inevitable result of tissue repair involving the interaction of fibroblasts, the ECM and cytokines. Increased vascular permeability and inflammation following burn injury may cause an increase in mast cells and stimulate the release of mast cell chymase from secretory granules (1,40). Results from the present study showed that in burn tissues, mast cell chymase contributed to the conversion of Ang II, the activation of TGF-β1 and the production of collagens I and III. Mast cell chymase is able to induce skin fibroblast proliferation (33,41), but the present study showed that mast cell chymase had no effect on fibroblast apotosis. The study indicated that mast cell chymase is conducive to wound healing.

In conclusion, in a hamster model of burn injuries, ketotifen, a mast cell membrane stabilizer, decreased the local concentration of Ang II, the expression levels of TGF-β1 and collagens I and III and the concentration of inflammatory marker IL-1β. These results suggest that mast cell chymase contributes to burn wound healing. Thus, chymase activity provides a promising future therapeutic target to accelerate wound healing.
